# Clinical application of ultrasound-guided core needle biopsy with multiple punches in the diagnosis of lymphoma

**DOI:** 10.1186/s12957-015-0537-2

**Published:** 2015-03-27

**Authors:** Ying He, Xiuzhen Ji, Yanggui Xie, Bosheng He, Xiaohong Xu, Xudong Chen, Qin Zhang

**Affiliations:** Department of Ultrasound, The Cancer Hospital of Nantong University, No. 30 Tongyang North Road, Pingchao Town, Tongzhou District, Nantong, 226361 China; Department of Ultrasound, The First Affiliated Hospital of Nantong University, No. 20 Xisi Road, Nantong, 226001 China; Department of Radiology, The Second Affiliated Hospital of Nantong University, No.6 Hai Er Xiang North Road, Nantong, Jiangsu 226001 P.R. China; Department of Oncology, The Cancer Hospital of Nantong University, No. 30 Tongyang North Road, Pingchao Town, Tongzhou District, Nantong, 226361 China; Department of Pathology, The Cancer Hospital of Nantong University, No. 30 Tongyang North Road, Pingchao Town, Tongzhou District, Nantong, 226361 China

**Keywords:** Lymphoma, Ultrasound-guided, Core needle biopsy, Multiple punches, Accuracy rate

## Abstract

**Background:**

The purpose of this study is to investigate the feasibility, accuracy, and limitations of ultrasound (US)-guided core needle biopsy (CNB) with multiple punches in the diagnosis of lymphoma in the whole body.

**Methods:**

From March 2007 to October 2013, US-guided CNB with multiple punches was performed by well-experienced radiologists in 110 patients (CNB group), and surgical biopsy was carried out in 95 patients (surgical group). The differences of accuracy rate between the two groups in the diagnosis of lymphoma and its subtypes were examined with Fisher’s exact test.

**Results:**

There were no statistical differences between the CNB group and the surgical group in the diagnostic accuracy rate of lymphoma, as well as its subtypes in superficial and deep masses. In addition, in the CNB group, there were no statistical differences between different lengths of lesions in the diagnosis accuracy rate of lymphoma and its subtypes.

**Conclusions:**

US-guided CNB with no less than three punches is an accurate, safe, minimally invasive, non-radiological, fast, and cost-effective method in the evaluation of lymphoma and its subtypes as compared with surgical approach. It should be considered as the acceptable alternative to surgical biopsy to obtain histopathological samples in the patients with suspected lymphoma.

## Background

Different subtypes of lymphoma vary in clinical manifestations, treatment, and prognosis; therefore, early diagnosis and histological classification are crucial for the assignment of therapeutic schedule [1]. In the past, confirmed diagnosis and classification of lymphoma mainly relied on surgical biopsy which not only increased the patients’ pains but also added up to their medical costs [2]. In recent years, minimally invasive techniques are gradually gaining recognition and have been widely used in the diagnoses of lymphoma.

Among the minimally invasive techniques, core needle biopsy (CNB) has been recognized as an alternative technique for diagnosing and subclassing the malignant lymphomas. This technique not only provides the architecture of the lymph node and sufficient tissue for further examinations such as immune phenotype, molecular genetics, and molecular biology as compared with fine needle biopsy (FNB) but also saves more than 75% of costs as compared with surgical biopsy [3]. Moreover, CNB is a non-radiological and real-time examination and may be the first initial approach chosen for people who cannot endure surgery, especially for feeble or older people. Therefore, CNB has gradually been accepted as an alternative method in the diagnosis of patients with lymphoma in the United States and some Western European countries because of its good applicability, safety, and high diagnostic rate [4-11].

It has been well established that CNB is effective and useful in the diagnosis of malignant lymphomas in many organs, such as head, neck, breast, thyroid, chest, and abdomen [11-17]. However, some studies merely focused on isolated localization such as superficial or deep masses or merely paid close attention to one or two organs. In addition, the influence on different lengths of lesions in the diagnosis accuracy of lymphoma and its subtypes by means of CNB is still not clear.

The purpose of this study was to investigate the feasibility, accuracy, and limitations of ultrasound (US)-guided CNB with multiple punches in the diagnosis of lymphoma in the whole body and evaluate its clinical value. The differences between CNB and surgical group in the diagnostic accuracy rate of lymphoma and its subtypes in superficial and deep masses of the whole body were compared, as well as the influence on different lengths of lesions in the diagnosis accuracy rate of lymphoma and its subtypes in CNB group.

## Methods

### Patients

A total of 205 patients underwent biopsy in the Department of Ultrasound, The Cancer Hospital of Nantong University for lymphoma lesion biopsy between March 2007 and October 2013 were enrolled. All patients were randomly assigned to US-guided CNB group and surgery group. All biopsies were done on an outpatient basis, unless inpatient medical supervision is needed. In the US-guided CNB group, there were 110 patients (aged 58.4 ± 17.3 years) consisting 60 males and 50 females. There were 62 cases of superficial masses (neck, axillary, groin, breast, vertical muscle, back) and 48 cases of deep masses (abdominal cavity, retroperitoneum). A total of 95 patients consisting 56 males and 39 females were assigned to the surgery group. The mean age was 58.9 ± 14.4 years. Surgery biopsies included 60 cases of superficial masses (neck, axillary, groin, breast, vertical muscle, back) and 35 cases of deep masses (abdominal cavity, retroperitoneum). However, two patients failed to be defined as subtypes of lymphoma after the first surgical biopsy. Six months later, they were confirmed as lymphoma and its subtypes in the second surgical biopsy.

Informed consents were obtained from all participants before CNB or surgery. Additionally, this study was approved by the Cancer Hospital of Nantong University medical ethics committees.

### Ultrasound-guided CNB

**Figure 1:**
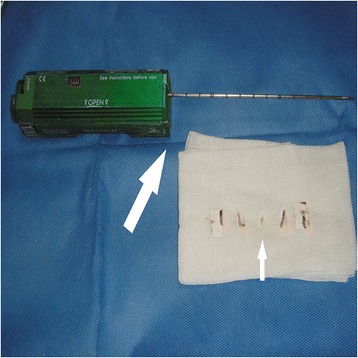
**Bard Magnum tissue-cutting needle and the third generation automatic biopsy gun (big arrow); tissue strips obtained with CNB (small arrow).**

**Figure 2:**
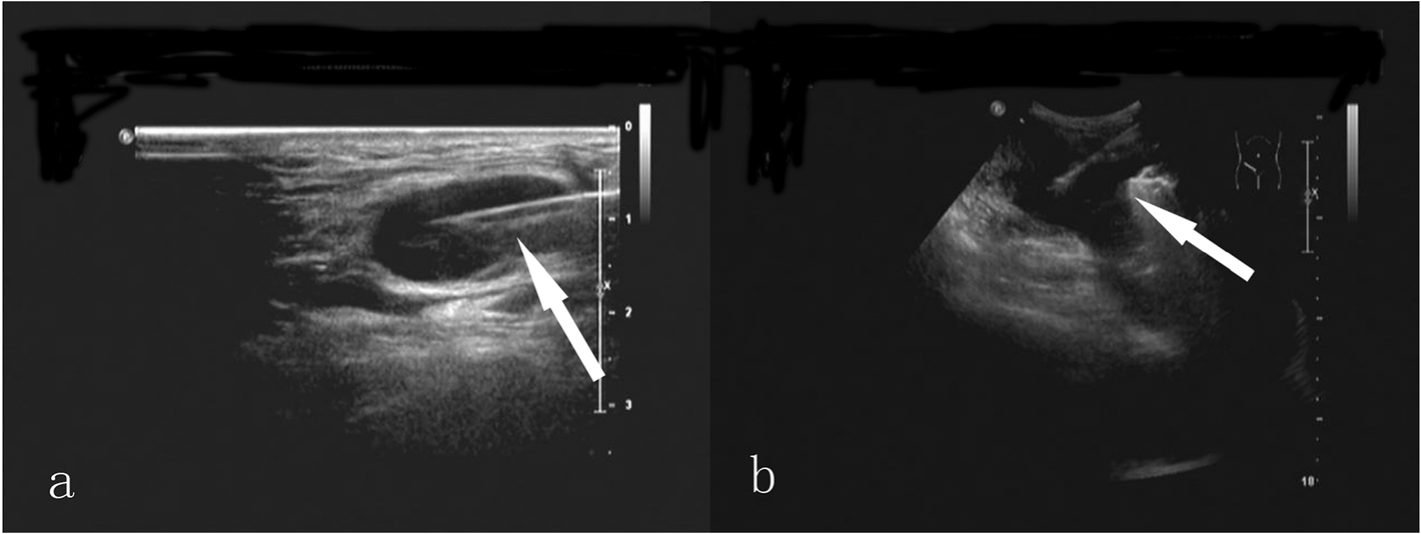
**Ultrasonograms of the needle tracts. (a)** The ultrasonogram of the needle tract in the superficial masses after ejection of biopsy gun (arrow); **(b)** the ultrasonogram of the needle tract in the deep masses after ejection of biopsy gun (arrow).

All biopsies were conducted under the supervision of color doppler ultrasonography (Philips IU-22, Amsterdam, The Netherlands) with probe frequencies of 2 to 5 MHz (low frequency) and 5 to 12 MHz (high frequency). The 14-gauge (14G) and 16-gauge (16G) cutting needles and the third generation automatic biopsy gun (Bard Magnum, Covington, GA, USA) (Figure 1) were used to the biopsies. The length of needle groove was 15 or 22 mm, depending upon the size of nodal and proximity of vessels [18]. The high-frequency probe-guided 14G core needle and low-frequency probe-guided 16G core needle were used to the biopsies of superficial and deep masses, respectively (Figure 2).

Normal coagulation screening tests, including prothrombin time (PT), platelet count, and partial thromboplastin time (PTT), were performed before biopsy [19]. If the results were confirmed to be normal, CNB would be performed to the patients. The routine US was carried out to investigate the shape, echo, size, and blood supply of the masses, and the relationships among the masses, surrounding organs, and great vessels, besides the medium length of the lesions, were recorded and compared between the two groups. All the biopsies were performed by well-experienced radiologists. The local anesthesia was administered using 2% lidocaine. Under the real-time supervision of US, the needle was inserted at the edge of the mass and the biopsy gun was instantaneously excited to collect at least three times in different parts of the mass (one needle in the center and two needles at the periphery of the mass). Intact tissue strips with a length of more than 0.5 cm were considered to be satisfactory samples.

**Figure 3:**
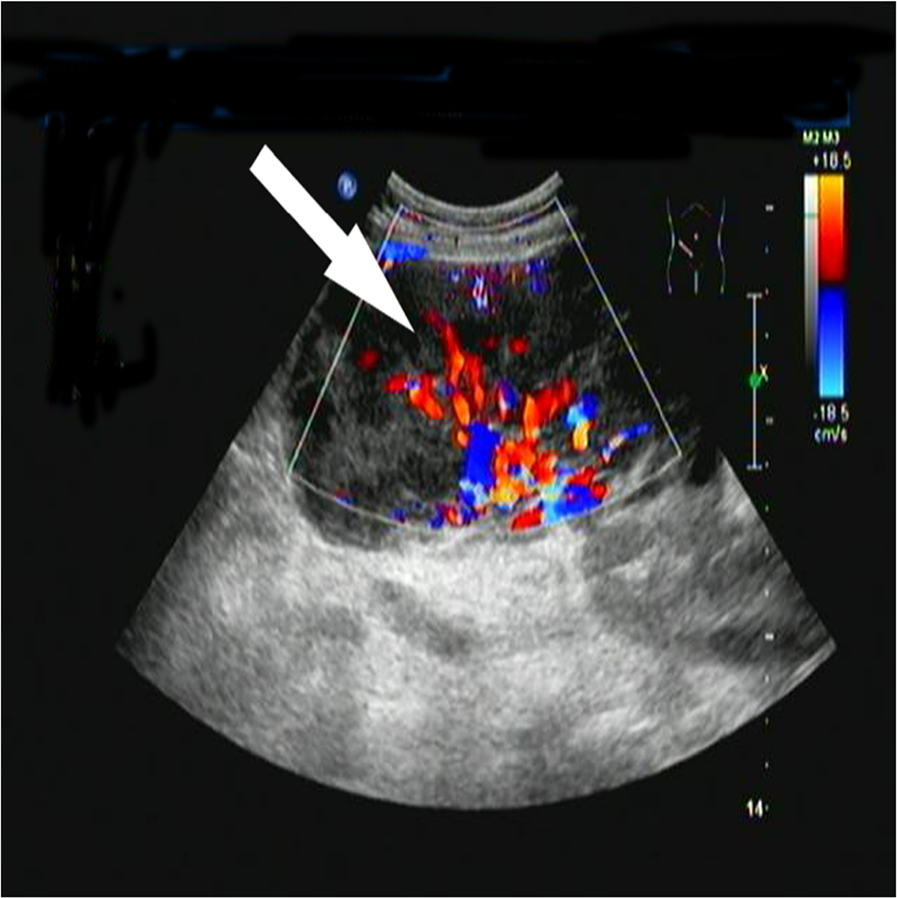
**CDFI shows the rich blood flow in the lesion (arrow).**

Different body positions were chosen according to different sites of the masses and the locations of samples. The sampling was collected with the help of surgical knife blade, especially for the tough skin. Moreover, the sampling points should be as close as possible to the body surface and avoid the necrotic areas. The samples should be harvested from hyper-vascular tissue (Figure 3). After CNB, the puncture points should be compressed sufficiently to avoid subcutaneous hematoma.

### Surgical biopsy

Adequate preoperative evaluation was done to master the indications, and normal coagulation screening tests were also assigned to the patients prior to the operation. If the results were confirmed to be normal, surgery would be allowed to perform. The surgical approach and biopsy range of masses were determined after a comprehensive analysis of clinical and imaging materials. All the surgical operation was performed by well-experienced surgeons. Excisional biopsy tissue should include the outer edge of the masses and hyper-vascular areas. The mass which was less than 3 cm should be wholly excised.

After the surgical biopsy, all patients in these two groups were kept under observation and then were returned to wards only if there was no sign or symptom of complications, such as bleeding. All patients were encouraged to call a doctor if they felt uncomfortable.

### Pathological diagnosis of lymphoma

After excision of the lesions, the final diagnosis for each case was established by secondary histopathology examination which was considered as a gold standard. All samples were placed in 10% formalin and embedded in paraffin. Serial sections (4 μm) were cut and stained with hematoxylin and eosin (HE). However, histologic examination which is utilized to reveal the morphology of lymphomas is now not adequate to distinguish the different categories of lymphoma. Molecular pathology techniques, such as immunohistochemistry (IHC), have been proven effective in diagnostic approaches to malignant lymphoma. Immunophenotyping to determine lineage and clonality using more than 80 different monoclonal antibodies is now supported to distinguish the different categories of lymphoma. Hence, IHC was performed to assess the immunophenotyping in our study. The appropriate selection of the specific monoclonal antibodies should be based on morphology and relevant clinical data to avoid pitfalls in the interpretation of IHC data. In our study, IHC was implemented using EnVision two-step method on a panel of antibodies [20]: leukocyte common antigen (LCA), cluster designation (CD)20, CD79a, CD10, Bcl-6, multiple myeloma oncogene (MUM)1, Ki-67, Bcl-2, Bcl-10, CD5, CyclinD1, CD23, anaplastic lymphoma kinase (ALK)-1, CD3, CD45RO, CD56, TIA-1, and CXCL13 (Beijing Zhongshan Jinqiao Biotechnology Corporation Limited). Pathological diagnosis was referred to WHO classification of lymphatic and hematopoietic tumors.

### Statistical analysis

Continuous variables were expressed as the mean ± standard deviation (SD). Statistical analysis was performed with the STATA10.0 statistical software package (STATA Corporation, College Station, TX, USA). The accuracy rate was examined with Fisher’s exact test. Findings with *P* < 0.05 were considered to be statistically different.

## Results and discussion

### CNB group

The medium length of the lesions was 7.3 cm (range 2.3 to 12.3 cm). Majority of the lesions were greater than 3 cm (84.5%, 93/110) (Table 1). During the puncture, three patients presented pains and one patient appeared minor hemorrhage (<60 ml). There was no serious complication in all cases. The medium length of tissue strips of each case was 1.5 cm (range 1.2 to 1.8 cm). The medium puncture number was 3.1 needles (range 2 to 6 needles) (Table 2). One patient was punctured for two needles due to local bleeding. The final pathological diagnosis included 2 cases of Hodgkin lymphoma (HL) and 108 cases of non-Hodgkin lymphoma (NHL) (Table 3). Diffuse large B cell lymphoma accounted for 77.3% (85/110). Eight patients failed to be defined as subtypes of lymphoma after the first CNB (including five patients undiagnosed as lymphoma). All the eight cases were assigned to further surgical biopsy to confirm the diagnosis (one case was performed two times) (Table 4).

**Table 1:** **Diagnostic accuracy of CNB in different lengths of lesions**

**Length**	**Number of cases**	**Diagnosis accuracy of lymphoma (%)**	**Diagnostic accuracy of subtypes (%)**
D ≤ 3	17	88.2 (15/17)	82.3 (14/17)
3 < D ≤ 5	34	97.1 (33/34)	94.1 (32/34)
D > 5	59	96.6 (57/59)	95.0 (56/59)

CNB, core needle biopsy; D, the length of llesions.

**Table 2:** **Number of punctures in CNB group**

**Number of punctures**	**Number of cases**	**Total number of punctures**
2	1	2
3	98	294
4	6	24
5	4	20
6	1	6
Total	110	346

CNB, core needle biopsy.

**Table 3:** **Lymphoma subtypes in CNB group and surgical group**

**Subtypes**	**Number of CNB group cases**	**Number of surgical group cases**
NHL	108 (98.2%)	94 (98.9%)
Diffuse large B cell lymphoma	85 (78.7%)	63 (67.0%)
Anaplastic large cell lymphoma	7 (6.5%)	3 (3.2%)
Mantle cell lymphoma	3 (2.8%)	8 (8.5%)
Follicular lymphoma	2 (1.9%)	5 (5.3%)
Small B cell lymphomas	2 (1.9%)	0 (0%)
Small lymphocytic lymphoma/leukemia	2 (1.9%)	1 (1.1%)
Nodal marginal zone B cell lymphoma	1 (0.9%)	1 (1.1%)
Lymphoblastic leukemia, T cell	1 (0.9%)	0 (0%)
Unspecified peripheral T cell lymphoma	1 (0.9%)	2 (2.1%)
Angioimmunoblastic T cell lymphoma	1 (0.9%)	0 (0%)
Burkitt’s lymphoma	1 (0.9%)	0 (0%)
B lymphocytic lymphoma/leukemia	1 (0.9%)	3 (3.2%)
Plasma cell differentiation large B cell lymphoma	1 (0.9%)	0 (0%)
B cell lymphoma of mucosa-associated lymphoid tissue	0 (0%)	5 (5.3%)
T-lymphoblastic lymphoma	0 (0%)	2 (2.1%)
NK/T cell lymphoma	0 (0%)	1 (1.1%)
HL	2 (1.8%)	1 (1.1%)
Hodgkin lymphoma nodular sclerosing type	2 (100%)	0 (0%)
Hodgkin lymphoma mixed cellularity type	0 (0%)	1 (100%)

CNB, core needle biopsy.

**Table 4:** **Eight cases undiagnosed as subtypes of lymphoma with CNB**

**Location**	**Pathological diagnosis with CNB**	**Pathological diagnosis with surgical biopsy**	**Remarks**
Abdominal cavity	Malignant tumor, poorly differentiated carcinoma	Diffuse large B cell lymphoma	5 years after gastric cancer operation
Abdominal cavity	Lymphoma	Diffuse large B cell lymphoma	
Left inguinal region	Lymphadenosis	Small lymphocytic lymphoma/leukemia	Hemorrhage
Right neck	Lymphadenosis heteromorphosis	Anaplastic large cell lymphoma	It is confirmed after the second surgery
Left armpit	Lymphadenosis heteromorphosis	Small B cell lymphomas	
Right armpit	Chronic inflammation accompanied with lymphadenosis	Angioimmunoblastic T cell lymphoma	
Left armpit	Lymphoma	Unspecified peripheral T cell lymphoma	
Right neck	Lymphoma	Nodal marginal zone B cell lymphoma	Rare type

CNB, core needle biopsy.

### Surgical group

During the procedure, there were 5 patients who had hemorrhage (100 to 200 ml) and 10 patients presented incision pain. The final pathological diagnosis included 1 case of HL and 94 cases of NHL (Table 3). Two cases failed to be diagnosed as lymphoma and its subtypes in the first surgical biopsy. They were confirmed to be follicular lymphoma and diffuse large B cell lymphoma after the secondary surgical biopsy. Among the 95 cases, one patient with retroperitoneal tumor presented hypoproteinemia a week after the surgical biopsy but was improved after symptomatic treatment. The surgical incision healed slowly in one patient who had diabetes. There were no serious complications in all cases.

### Comparison of diagnosis accuracy rate between the two groups

There was no statistical difference between CNB and surgical group in the diagnostic accuracy rate of lymphoma and its subtypes in superficial (*P* = 0.68; *P* = 0.273) and deep masses (*P* = 1; *P* = 0.506), respectively (Table 5). In the CNB group, there was no statistical difference between different lengths of masses in the diagnostic accuracy rate of lymphoma and its subtypes.

**Table 5:** **Diagnostic accuracy of lymphoma and its subtypes between CNB and surgical group in superficial and deep masses**

**Location**	**Diagnosis**	**CNB group**	**Surgical group**	***P***
Superficial masses	Lymphoma	93.5 (58/62)	96.6 (58/60)	0.68
	Subtypes	90.3 (56/62)	96.6 (58/60)	0.273
Deep masses	Lymphoma	97.9 (47/48)	100 (35/35)	1
	Subtypes	95.8 (46/48)	100 (35/35)	0.506
Total	Lymphoma	95.4 (105/110)	97.9 (96/98)	0.454
	Subtypes	92.7 (102/110)	97.9 (96/98)	0.11

CNB, core needle biopsy.

### Discussion

In our present study, we investigated the feasibility, accuracy, and limitations of CNB with multiple punches in the diagnosis of lymphoma in the whole body. The results suggested that US-guided CNB with no less than three punches was an accurate, safe, minimally invasive, non-radiological, fast, and cost-effective method in the evaluation of lymphoma and its subtypes compared with surgical approach.

US-guided CNB is a non-radiological and real-time examination, which can provide blood flow information of lesions ensuring the accuracy of sampling. Moreover, the obtained sample is intact, and the cells are not compressed which is helpful for pathological examination [21]. In addition, CNB enables us to streamline patient care and reduce operating time and expense compared with surgery [21]. Previous study indicated that the success rate of CNB was significantly higher than FNB (37.6%) [22] and CT-guided CNB (71.5%) [23]. A large number of studies have documented that CNB is an effective and reliable procedure with a high diagnostic yield for lymphoma [9,24,25].In our studies, we evaluated the diagnostic accuracy of lymphoma and its subtypes in superficial and deep masses using CNB and surgery. The results showed that there was no statistical difference (*P* = 0.273, and *P* = 0.506, respectively), indicating that CNB was not only suited for deep masses but also for superficial masses. In the past, surgery was advocated for superficial masses; however, our study provided some evidence to back up CNB for diagnosing superficial masses. What is more, we also found that there was no significant difference between different lengths of lesions in the diagnostic accuracy rate of lymphoma and its subtypes.

Hemorrhage is the most common complication of needle biopsy, and the incidence is 0.8% to 3.0% [26,27]. In our study, there was only one patient who had minor hemorrhage (<60 ml) and three patients presented pains. In addition, there were no serious complications in all cases. Whereas, there were 5 patients who had hemorrhage (100 to 200 ml) and 10 patients presented incision pain in the surgery group. On the whole, the US-guided CNB reduced the complications as compared with surgical biopsy.

Although percutaneous US-guided CNB is currently the best way to obtain positive histopathological diagnosis of lymphoma under the non-surgical conditions, several points should be noted. First of all, CNB should be performed by well-experienced radiologists to acquire adequate sample. Secondly, sufficient and high-quality sample volume may be the key factors affecting the accuracy rate of CNB [28,29]. Therefore, sampling sites should be chosen in or closed to the rich blood supply areas and in the periphery of the lesion. Thirdly, US-guided CNB should be used for the lesions visualized clearly by ultrasound; however, for the lesions which could not be displayed clearly by US or negative results of CNB, local surgical biopsy should be performed. Fourthly, the application of 14G and 16G needles and automatic biopsy gun also should be noteworthy. In our study, 14G and 16G were used to the biopsies of superficial and deep masses, respectively. The choice of biopsy needle size mainly depends on the size and localization of lesions [14]. Generally speaking, large core needles (usually 14G) are utilized to superficial biopsies, whereas smaller needles (18 or 16G) are performed on deep biopsies [30]. The last point is multi-point sampling. Usually, lymphoma is characterized by multifocal in origin; therefore, multi-point sampling should be conducted to acquire effective and adequate tissue. Bolivar *et al*. [31] suggested that each lesion should be punctured for no less than three needles in the diagnostic value of US-guided 14G CNB in non-palpable suspicious breast lesions. In line with their study, we also found that no less than three punches may be effective for CNB in diagnosing lymphoma in our study.

In the CNB group, eight patients failed to be defined as subtypes of lymphoma after the first CNB (including five patients undiagnosed as lymphoma). The eight patients then underwent surgical biopsy to confirm the final diagnosis and subtypes of lymphoma. Of the five patients undiagnosed as lymphoma, one patient was confirmed finally to have anaplastic large cell lymphoma. The reasons for the failure of diagnosis with CNB may be relevant to reactive hyperplasia in the sampling areas or the sample was not a representative of the characteristics of lymphoma. One patient had minor hemorrhage and was punctured with only two needles resulting in the reduction of sample volume. For the other one case, the tumor was the largest among all patients enrolled in this study and the sample was probably punctured in necrotic tissues. In the three cases failed to be confirmed to be subtypes of lymphoma, one case was a rare type (nodal marginal zone B cell lymphoma) and the diagnosis may be difficult, and the other two cases may be relevant to less sample volume.

However, there were several limitations in our study. The lesions were only divided into superficial and deep masses; the specific location of the enlarged lymph nodes was not taken into consideration. The lesions which were not displayed clearly by US were not enrolled in the CNB group. All of these may lead to biased results. In addition, our study did not intensively investigate the quality control factors of the golden standard (pathological diagnosis).

## Conclusions

In summary, US-guided CNB with no less than three punches is an accurate, safe, minimally invasive, convenient, fast, and low-cost method in the evaluation of lymphoma and its subtypes. It should be used as the acceptable alternative to surgical biopsy to obtain histopathological samples in the patients with suspected lymphoma.

## Abbreviations

CNB: core needle biopsy
FNB: fine needle biopsy
HE: hematoxylin and eosin
HL: Hodgkin lymphoma
IHC: immunohistochemistry
NHL: non-Hodgkin lymphoma
PT: prothrombintime
PTT: partial thromboplastin time
SD: standard deviation.
